# Outcome Analysis of Surgical Timing in Pediatric Orbital Trapdoor Fracture with Different Entrapment Contents: A Retrospective Study

**DOI:** 10.3390/children9030398

**Published:** 2022-03-11

**Authors:** Pei-Ju Hsieh, Han-Tsung Liao

**Affiliations:** 1 Division of Traumatic Plastic Surgery, Department of Plastic and Reconstructive Surgery, Craniofacial Research Center, Chang Gung Memorial Hospital at LinKou, Chang Gung University College of Medicine, Taoyuan City 333, Taiwan; b101103094@tmu.edu.tw; 2 College of Medicine, Chang Gung University, Taoyuan City 333, Taiwan; 3 Department of Plastic Surgery, Xiamen Chang Gung Hospital, Xiamen 361000, China

**Keywords:** adolescents, children, pediatric orbital fracture, orbital trapdoor fracture

## Abstract

Orbital trapdoor fracture occurs more commonly in pediatric patients, and previous studies suggested early intervention for a better outcome. However, there is no consensus on the appropriate timing of emergent intervention due to the insufficient cases reported. In the current retrospective study, we compared the outcomes of patient groups with different time intervals from injury to surgical intervention and entrapment content. Twenty-three patients who underwent surgery for trapdoor fracture between January 2001 and September 2018 at Chang Gung Memorial Hospital were enrolled. There was no significant difference in diplopia and extraocular muscle (EOM) movement recovery rate in patients who underwent surgery within three days and those over three days. However, among the patients with an interval to surgery of over three days, those with muscle entrapment required a longer period of time to recover from EOM movement restriction (*p* = 0.03) and diplopia (*p* = 0.03) than those with soft tissue entrapment. Regardless of time interval to surgery, patients with muscle entrapment took longer time to recover from EOM movement restriction (*p* = 0.036) and diplopia (*p* = 0.042) and had the trend of a worse EOM recovery rate compared to patients with soft tissue entrapment. Hence, we suggested that orbital trapdoor fractures with rectus muscle entrapment should be promptly managed for faster recovery.

## 1. Introduction

Orbital trapdoor fracture occurs more frequently in patient under 18 years old. Due to the inherent elasticity of facial bone, the displaced orbital wall recoils back and traps the soft tissue or rectus muscle, causing extraocular muscle (EOM) movement restriction and diplopia. The injury may also induce oculocardiac reflex by traction on EOM, causing bradycardia, nausea, and syncope [1]. To remove these acute symptoms, releasing the entrapment content is significantly effective. However, not every patient fully recovers from EOM movement restriction and diplopia after the operation. The mechanism remains unclear, but most authors have agreed that the entrapment induces muscle incarceration and causes irreversible muscle fibrosis [2,3,4,5].

Previous studies have advocated early intervention to shorten the duration of muscle ischemia [1,6,7,8]. Moreover, recent studies have also shown that entrapment content significantly influenced the outcome of trapdoor fracture [5,9,10]. However, there is no consensus on the appropriate timing and indication of urgent surgical intervention due to the low incidence of trapdoor fractures and the small sample size.

In our retrospective study, we compared and analyzed the outcome of patient groups with different time intervals from injury to surgical intervention and content of entrapment (muscle versus soft tissue).

## 2. Materials and Methods

### 2.1. Design and Participants

We recruited patients under 18 with pure orbital wall fractures who underwent surgery at Chang Gung Memorial Hospital, Taiwan, between January 2001 to September 2018.

### 2.2. Procedures and Measures

A total of 23 patients had orbital trapdoor fractures. Preoperative computed tomography (CT) images and clinical symptoms of restricted EOM movement and diplopia confirmed the trapdoor fracture diagnosis. Patients with incomplete clinical and radiological evidence or a postoperative follow-up duration of fewer than six months were excluded. Cause of fractures, entrapment content, time interval from injury to surgery, the severity of EOM movement restriction and diplopia before and after surgery, and time interval from surgery to full recovery were recorded. Measurements of EOM movement were based on a numeric scale, with 3 representing no limitation and 0 representing no movement in one direction of gaze (Table 1). We evaluated the preoperative EOM movement, and the patient underwent an operation on the same day.

As for surgical intervention, we performed a forced duction test after general anesthesia before incision. The surgery started with a transconjunctival or subciliary approach to the orbital wall. After dissection and exposure of the orbital wall, the entrapped orbital contents were gently released from the fracture site. The fractured wall was patched with MEDPOR (Stryker, Kalamazoo, MI, USA) to prevent recurrent herniation or entrapment. Forced duction tests were performed again at the end of surgery to confirm the complete release of the entrapped tissue. We followed up with the patients every month for over half a year and recorded the latest EOM score and the results of examinations for diplopia. We took the worst preoperative EOM score for analysis regardless of the direction. We then took the postoperative EOM score of the same direction as the preoperative EOM score for comparison.

### 2.3. Statistical Analysis

Data were analyzed using SPSS V.19.0 (IBM, Portsmouth, UK). The data analysis included descriptive statistics, Fisher’s Exact Test, and Mann–Whitney U Test. Significance was established at 0.05. We used Mann–Whitney U Test to analyze the score of EOM movement and time interval from injury to full recovery of both EOM movement restriction and diplopia. Fisher’s Exact Test is used for the recovery rate of both EOM movement restriction and diplopia.

## 3. Results

Of the 23 patients enrolled, the average age was 10.78 years old (SD: 3.57), ranging from 6–18. The male and female ratio was 17:6. The most common cause of injury was falling (30.4%), followed by assault (26.1%), and blunt trauma (21.7%). Among the fracture sites, twenty were at the orbital floor, and three were at the medial orbital wall. As for entrapment content, 12 patients had rectus muscle entrapment, with the rest having pure soft tissue entrapment. The average time interval from injury to operation was 12.95 ± 16.8 days, with 6.0 ± 8.8 and 20.5 ± 20 days in muscle and soft tissue trapdoor fracture, respectively. The overall recovery rates of EOM movement restriction and diplopia were 87.0% (SD: 33.68%) and 73.91% (SD: 43.91%), respectively. The average time interval to full recovery was 174.47 (SD: 244.91) days for the symptom of EOM movement restrictions and 293.75 (SD: 537.09) days for diplopia (Table 2).

We initially divided the patients into two groups—surgical intervention within three days (Early) and over three days (Late). There was no significant difference between the two groups in preoperative EOM movement, diplopia, postoperative symptoms, and recovery time (Table 3). We then stratified the patient group with surgical time interval by entrapment content. There was no significant difference between the muscle and soft tissue entrapment group which performed surgery within three days in post-op EOM movement, EOM movement full recovery rate, persistent diplopia rate, and interval to full recovery of EOM movement and diplopia. Patients with muscle entrapment showed worse preoperative EOM movement than those with soft tissue entrapment at surgical timing of more than three days (*p* = 0.026). There was no significant difference in the recovery rate in EOM movement restriction and diplopia between the two types of entrapment content, but the subgroup with muscle entrapment took significantly longer to recover from both EOM movement restriction (*p* = 0.030) and diplopia (*p* = 0.030) at surgical timing of more than three days (Table 4).

Furthermore, we divided the patients by their entrapment content—rectus muscle versus pure soft tissue. The patients with muscle entrapment had more serious preoperative EOM movement restriction (*p* = 0.002) and needed a longer time for EOM movement (*p* = 0.036) and diplopia (*p* = 0.042) recovery, significantly. (Table 5) We also divided each group into early and late surgical interventions. Although the trends of worse recovery rate and longer recovery time from EOM movement restriction and diplopia were found, there were no significant differences between subgroups in both types of entrapment content (Table 6).

## 4. Discussion

Due to the inherited elasticity of bone, pediatric patients encountering a blunt injury have a higher rate of trapdoor fractures than blowout fractures. The displaced bone would transiently snap back to its original position and impinge on the herniated tissue, causing the lower portion of extraocular tissue to be incarcerated. The incidence of entrapment in pure orbital wall fracture in pediatric patients was approximately 5.8% [5], and the age of patients ranged from 6–16 years old [2,11].

With a longer duration of incarceration, there is a higher rate of persistent diplopia. The exact mechanisms of persistent diplopia are still under debate, but it is mostly accepted that the trapped tissue would undergo ischemia and result in irreversible fibrosis and scarring [3,4]. Therefore, early surgical intervention for pediatric trapdoor fracture should be encouraged to better recover from EOM movement restriction and diplopia. As opposed to the traditional management of a 2-week waiting period of observation for a common blowout fracture, Jordan et al. [12] presented a case series and found that patients with intervention over two weeks had no full recovery of EOM movement restriction. In a retrospective study presented by Grant et al. [6], 19 patients with trapdoor fracture were enrolled and segregated into two groups by the overall means of time interval to intervention—5 days. They found a negative correlation between time interval and degree of EOM movement after releasing the entrapped content in the late intervention group (>5 days). Neinstein et al. [7] analyzed their participants retrospectively; the mean of time to surgery in the patients with full return of EOM function was 6.4 days compared with 14.2 days in those with residual diplopia. Yoon et al. [8] conducted a study in which patients were divided into three groups by intervention at 0–5 days, 5–14 days, and over 14 days. In the first month after surgery, early intervention within five days resulted in the greatest reduction of EOM movement limitation. Yang et al. [10] investigated patients with shorter surgical intervention and segregated them into three groups by surgical timing of fewer than 24 h, 24–72 h, and over 72 h. The authors found no significant differences in recovery rate and interval to full recovery between the three groups. For general trapdoor fracture, surgical timing within 3–7 days seems to bring a better recovery rate and shorter interval to full recovery.

Recent studies have mentioned that the entrapped content and type of fracture may affect the initial severity of diplopia and EOM movement restriction as well as the post-operatively outcomes. In the study by Gerbino et al. [2], they analyzed the outcome of patients with different extents of fracture bone displacement. With intervention performed within 24 h, in patients with minor bone fragment displaced, the tissue was trapped more tightly and had a higher incidence of residual diplopia. As for entrapment content, patients with rectus muscle tended to have more severe preoperative EOM movement restriction. Su et al. [9] performed a study on patients with delayed surgical intervention. They divided the patients by entrapment content and stratified them by their severity of preoperative EOM movement limitation. While the extent of initial EOM movement restriction did not affect the recovery rate and time interval to full recovery, the patient subgroup with rectus muscle entrapment took a significantly longer time to full recovery. Besides surgical timing, other factors that affected the preoperative symptoms of trapdoor fracture were proved to have some influences on the outcome [6,10]. Therefore, surgical timing could be adjusted according to the patient’s extent of injury and entrapment contents. Our study also demonstrated that muscle entrapment leads to delayed recovery of EOM movements and diplopia.

Early diagnosis of trapdoor fracture may be decisive for the outcome for pediatric patients. According to previous studies, indications to surgical intervention should not rely on merely radiographic evidence of entrapment [1,2,8,13]. Contents of entrapment would occasionally be too subtle to be recognized in CT images, and the concordance rate to operative findings was reported to be 50% in the pediatric population [9,13]. Thin-sliced CT images and missing rectus signs were recommended for entrapment content discovery. Yet, correlation to severe clinical evidence is considered a critical indication of surgery [13,14]. Since children may be difficult to approach during an emergency due to the inability to describe their discomfort and poor cooperation, physical examination plays a significant role in diagnosis. External clinical signs such as ecchymosis, edema, and enophthalmos are relatively minimal in children compared to adults. Instead, EOM movement restriction could indicate a high possibility of muscle entrapment even without radiographic findings. Moreover, muscle entrapment may also bring oculocardiac reflex (OCR), which is more commonly seen in children and adolescents [15]. Arrhythmia caused by OCR may put pediatric patients in danger but could be easily relieved by releasing the injured rectus muscle. Kim et al. [1] found a strong association between nausea and vomiting and extraocular muscle entrapment. In other words, severe OCR should be considered a sign of muscle entrapment and taken as one of the indications for immediate surgery [16,17,18]. Compared to pure soft tissue entrapment, muscle entrapment with delayed intervention would need a longer time to recover and higher risks for persistent diplopia and fatal complications. Therefore, even though the exact cut-off timing for intervention is still under debate, the urgency of an operation could be considered according to the symptom severity.

Limitations of this study include its retrospective nature and relatively small sample size. The number of participants in the early intervention group was insufficient for statistical validity as our institute is classified as a tertiary medical center and our patients are mostly composed of referral from other local hospitals which leads to delayed visit and management. Furthermore, most of the recruited patients with pure soft tissue entrapment in this study visited our hospital more than three days after injury because they were unaware of the severity due to mild diplopia or EOM movement restriction. Moreover, some children did not receive surgery because of mild symptoms and signs. However, the surgical timing still remains vital as previous studies have recommended that patients with soft tissue entrapment should be treated as having muscle entrapment when they present restriction in EOM [9].

## 5. Conclusions

In conclusion, we recommend early intervention for patients with rectus muscle entrapment and severe symptoms of trapdoor fracture. Although an appropriate timing remained unknown, our study showed that patients with muscle entrapment would have a shorter interval to full recovery if receiving surgical intervention within three days. Although the recovery rate is not related to either surgical timing or entrapment content, early intervention should be conducted to lower the risks of potentially fatal complications, improve the outcomes, and enhance faster recovery.

## Figures and Tables

**Table 1:** The numeric scale for the measurement of EOM movement.

Score	Definition
3	No limitation in one direction of gaze
2	Active movement range > 50% of primary position to the edge of conjunctiva without full motion in one direction of gaze
1	Active movement range < 50% of primary position to the edge of conjunctiva without full motion in one direction of gaze
0	No movement in one direction of gaze

**Table 2:** Patient Characteristics.

Patient Characteristics (*n* = 23)	Mean (SD)	
Age (Y)	10.78 (±3.57)	
Gender (M:F)	17:6	
Side (Right: Left)	15:11	
Fracture Site (Orbital Floor: Medial Wall)	20:3	
Entrapment content (Muscle: Soft tissue)	12:11	
Injury Mechanism (n)	Fall: 7	MVA: 3
	Assault: 6	Sports: 2
	Blunt trauma: 5	
Time Interval from Injury to Intervention (Days)	12.95 (±16.84)	
	Muscle: 6.03 (±8.79)	
	Soft Tissue: 20.50 (±19.99)	
Pre-OP EOM movement Score	0.90 (±1.07)	
	Muscle: 0.25 (±0.60)	
	Soft Tissue: 1.59 (±1.04)	
Post-OP EOM movement Score	2.80 (±0.64)	
Improvement in EOM movement restriction	1.9 (±1.1)	
Full Recovery Rate of EOM movement restriction	87.0% (±33.68%)	
Pre-OP Diplopia (percentage)	87.0% (±33.68%)	
Post-OP Diplopia (percentage)	26.09% (±43.91%)	
Full Recovery Rate of Diplopia	73.91% (±43.91%)	
Interval to full recovery of EOM movement restriction (Days)	174.47 (±244.91)	
Interval to full recovery of Diplopia (Days)	293.75 (±537.09)	

Pre-OP means preoperative; Post-OP means postoperative; EOM means extraocular muscle.

**Table 3:** Comparison of functional outcome by time interval to surgical intervention.

Time Interval (Days)	Pre-OP EOM Movement	Pre-OP Diplopia	Post-OP EOM Movement	EOM Movement Full Recovery Rate	Interval to Full EOM Movement Recovery	Persistent Diplopia	Interval to Diplopia Recovery
≤3 Days (*n* = 7)	0.21 ± 0.36	71.43 ± 45.18%	2.86 ± 0.36	85.71 ± 34.99%	84.07 ± 54.68	14.29% ± 34.99%	91.06 ± 51.80
>3 Days (*n* = 16)	1.19 ± 1.18	93.75 ± 24.21%	2.81 ± 0.73	87.50 ± 33.07%	214.01 ± 291.70	31.25% ± 46.35%	382.42 ± 643.04
*p*-Value	*p* = 0.082	*p* = 0.209	*p* = 0.585	*p* = 1.000	*p* = 0.624	*p* = 0.621	*p* = 0.535

Pre-OP means preoperative; Post-OP means postoperative; EOM means extraocular muscle.

**Table 4:** Comparison of functional outcome by surgical time interval and subgroups of entrapment contents.

Entrapment Content		Pre-OP EOM Movement	Pre-OP Diplopia	Post-OP EOM Movement	EOM Movement Full Recovery Rate	Interval to Full EOM Movement Recovery	Persistent Diplopia	Interval to Diplopia Recovery
≤3 Day (*n* = 7)	Muscle (*n* = 6):	0.17 ± 0.37	83.33 ± 37.27%	2.83 ± 0.37	83.33 ± 37.27%	96.46 ± 49.13	83.33 ± 37.27%	104.62 ± 42.94
	Soft Tissue (*n* = 1):	0.50	100.00%	3.00	100.00%	9.72	0.00%	9.72
*p*-Value		*p* = 0.334	*p* = 0.286	*p* = 0.683	*p* = 1.000	*p* = 0.699	*p* = 1.000	*p* = 0.699
>3 Day (*n* = 16)	Muscle (*n* = 6):	0.33 ± 0.75	83.33 ± 37.27%	2.50 ± 1.12	66.67 ± 47.14%	399.51 ± 358.18	50.00 ± 50.00%	751.26 ± 832.75
	Soft Tissue (*n* = 10):	1.70 ± 1.03	90.91 ± 0.29%	3.00	100.00%	102.72 ± 132.78	20.00 ± 40.00%	161.11 ± 271.22
*p*-Value		*p* = 0.026	*p* = 0.375	*p* = 0.197	*p* = 0. 125	*p* = 0.030	*p* = 0.299	*p* = 0.030

**Table 5:** Comparison of functional outcome by entrapment contents.

Entrapment Content	Pre-OP EOM Movement	Pre-OP Diplopia	Post-OP EOM Movement	EOM Movement Full Recovery Rate	Interval to Full EOM Movement Recovery	Persistent Diplopia	Interval to Diplopia Recovery
Muscle (*n* = 12)	0.25 ± 0.60	83.33 ± 37.27%	2.67 ± 0.85	75.00 ± 43.30%	247.99 ± 297.17	66.67 ± 47.14%	541.78 ± 742.00
Soft Tissue (*n* = 11)	1.59 ± 1.04	90.91 ± 28.75%	3.0	100.00%	94.26 ± 129.39	81.82 ± 38.57%	147.35 ± 262.23
*p*-Value	*p* = 0.002	*p* = 1.000	*p* = 0.166	*p* = 0.217	*p* = 0.036	*p* = 0.640	*p* = 0.042

**Table 6:** Comparison of functional outcome by entrapment contains and subgroup of surgical timing interval.

Time Interval (Days)		Pre-OP EOM Movement	Pre-OP Diplopia	Post-OP EOM Movement	EOM Movement Full Recovery Rate	Interval to Full EOM Movement Recovery	Persistent Diplopia	Interval to Diplopia Recovery
Muscle (*n* = 12)	≤3 Days (*n* = 6):	0.17 ± 0.37	83.33 ± 37.27%	2.83 ± 0.37	83.33 ± 37.27%	96.46 ± 49.13	83.33 ± 37.27%	104.62 ± 42.94
	>3 Days (*n* = 6):	0.33 ± 0.75	83.33 ± 37.27%	2.50 ± 1.12	66.67 ± 47.14%	399.51 ± 358.18	50.00 ± 50.00%	751.26 ± 832.75
*p*-Value		*p* = 0.902	*p* = 1.000	*p* = 0.902	*p* = 1.000	*p* = 0.078	*p* = 0.545	*p* = 0.078
Soft Tissue (*n* = 11)	≤3 Days (*n* = 1):	0.50	100.00%	3.00	100.00%	9.72	0.00%	9.72
	>3 Days (*n* = 10):	1.70 ± 1.03	90.91 ± 0.29%	3.00	100.00%	102.72 ± 132.78	20.00 ± 40.00%	161.11 ± 271.22
*p*-Value		*p* = 0.336	*p* = 0.091	-	-	*p* = 0.527	*p* = 1.000	*p* = 0.343

## Data Availability

The data that support the findings of this study are available on request from the corresponding author. The data are not publicly available due to privacy and ethical restrictions.
